# Transapical Approach for Mitral Valve Repair during Insertion of a Left Ventricular Assist Device

**DOI:** 10.1155/2013/925310

**Published:** 2013-06-26

**Authors:** Mark J. Russo, Aurelie Merlo, Elizabeth M. Johnson, Shahab Akhter, Sean McCarney, Jennifer Steiman, Allen Anderson, Valluvan Jeevanandam

**Affiliations:** ^1^Section of Cardiac and Thoracic Surgery, Department of Surgery, University of Chicago, Chicago, IL 60637, USA; ^2^Department of Cardiothoracic Surgery, Barnabas Health Heart Centers, Newark Beth Israel Medical Center, 201 Lyons Avenue, Suite G5, Newark, NJ 07112, USA; ^3^Section of Cardiology, Department of Medicine, University of Chicago, Chicago, IL 60637, USA

## Abstract

*Background*. Severe mitral regurgitation (MR) is common in patients who are undergoing insertion of a left ventricular assist device (LVAD). This study analyzes the outcomes of a transapical approach for edge-to-edge repair of the mitral valve during insertion of a left ventricular assist device in 19 patients with MR. *Methods*. This retrospective study includes 19 patients who were implanted between March 21, 2011, and August 31, 2011, at the University of Chicago. Clinical data include preoperative ejection fraction, post- and preoperative pulmonary arterial pressures, cardiopulmonary bypass time, post- and preoperative mitral regurgitation severity, endotracheal CO2, and LVAD pulse index. *Results*. All of the 19 patients had a reduction in mitral regurgitation. Fourteen of the 19 patients had at least a three-point reduction in MR severity. The average postoperative pulmonary arterial pressure (PAP) decreased after the surgical procedure from 44/22 ± 14/5 mmHg to 57/28 ± 9/5 mmHg. Average CPB time was 128 ± 27 minutes. Average length-of-stay (LOS) was 21 ± 10 days. *Conclusions*. Concomitant MV repair using a transapical approach is advantageous for this small cohort of patients. The surgical procedure is less complex and has a shorter CPB time and LOS, and all of the patients demonstrated significant improvement in postoperative MR and moderate improvement in PAP.

## 1. Introduction

As a consequence of left ventricular remodeling, severe mitral regurgitation (MR) is common in patients with end-stage heart failure who are undergoing insertion of a left ventricular assist device (LVAD). This valvular related pathology often develops in the absence of structural mitral valve abnormalities. Instead, regurgitation develops secondary to left ventricular cavity enlargement and/or increased ventricular sphericity with annular dilation [1]. 

Concomitant mitral valve repair during LVAD insertion increases the complexity of the operation due to the need for additional dissection and incisions in the heart, bicaval cannulation, and prolonged cardiopulmonary bypass times. Edge-to-edge repair, developed by Alfieri and associates, has been shown to be a fast and reliable method of mitral valve repair in appropriate patients [2, 3]. Here, we describe a transapical approach for edge-to-edge repair of the mitral valve during insertion of a left ventricular assist device in 19 patients with MR secondary to left ventricular (LV) dysfunction.

## 2. Material and Methods

### 2.1. Technique

**Figure 1:**
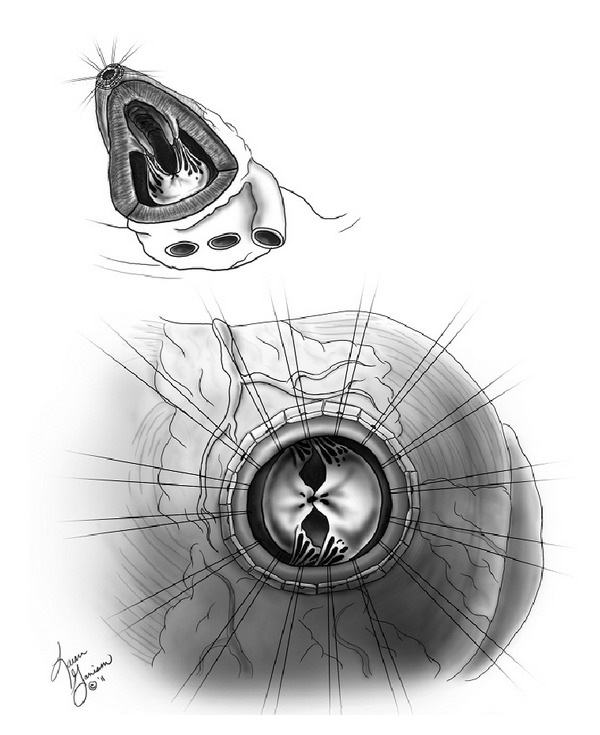
Apical sutures placed with Teflon pledgets in a horizontal mattress fashion.

The degree of MR is evaluated preoperatively by echocardiography and angiography. In the operating room, the mitral valve is again assessed preoperatively with transesophageal echocardiography (TEE). After a median sternotomy, preparation is made for initiation of cardiopulmonary bypass requiring only a single right atrial cannula for venous outflow and aortic cannula for arterial inflow. Cardiopulmonary bypass (CPB) is initiated and normothermic conditions are maintained. A left ventricular vent is placed via the right superior pulmonary vein. The heart is lifted out of the chest, exposing the LV apex. The apical coring knife is then utilized approximately 1.5 cm lateral to the left anterior descending artery in the apical dimple. The left ventricle is inspected for thrombus. Apical sutures are then placed with large Teflon felt pledgets in a horizontal mattress fashion (Figure 1). 

The mitral valve is then accessed through the apical incision. To better expose the mitral valve, a malleable retractor is used to retract the septum and a floppy sucker is passed between the anterior and posterior leaflet of the valve. The midpoint (equidistant from the two commissures) of the free edge of the anterior leaflet is identified, and the posterior leaflet is exposed. The midpoints of P2 and A2 are then sutured together using a 4-0 Prolene mattress suture buttressed with a felt pledget. The suture is placed approximately 5 mm from the free edge of the leaflets. The LVAD is then connected and secured in the standard fashion by passing sutures through the apical connector sewing ring and seating the apical connector in the LV apex. The LVAD is then attached to the apical connector and secured. The LVAD is positioned in the pericardium (HVAD, HeartWare, Framingham, MA) or preperitoneal pocket (Heartmate II, Thoratec Corp., Pleasanton, CA), and the driveline is tunneled and brought out in the right upper quadrant.

A partial occlusion clamp is then placed on the ascending aorta and an aortotomy is created. The distal anastomosis is then constructed between the outflow graft and the ascending aorta in an end to side fashion using running 4-0 Prolene suture. The outflow graft is then clamped and the partial occlusion clamp is released. Meticulous hemostasis is assured at the suture line. The outflow graft is then secured to the assist device. After deairing maneuvers, full VAD support is initiated and the patient is weaned from CPB. Transesophageal echo is then used to assess the degree of MR, mitral stenosis (MS), and ventricular function.

### 2.2. Data Collection

The study included 19 consecutive patients who underwent LVAD implantation with concomitant transapical edge-to-edge mitral valve repair between March 21, 2011, and August 31, 2011, at the University of Chicago. All data was collected retrospectively, and use of the data is consistent with the regulations of the University of Chicago's Institutional Review Board. Demographic and clinical characteristics are included in Table 1. Clinical data include preoperative ejection fraction, post- and preoperative pulmonary arterial pressures (PAP), cardiopulmonary bypass time, post- and preoperative mitral regurgitation severity, endotracheal CO2, and LVAD pulse index. 

## 3. Results

Relevant preoperative and postoperative clinical data are included in Table 2. Each of the 19 patients in this series experienced an improvement from moderate-to-severe MR to trace/trivial-to-mild MR. Sixteen (84.2%) of the 19 patients left the OR with 0 or level 1+ MR, and 14 (73.7%) of the 19 patients experienced at least a 3rd grade reduction in MR severity. None of the patients had measurable mitral valve stenosis. Further, there was a measureable decrease in postoperative PAPs. Before the LVAD insertion and MV repair, the mean PAP of 57/28 ± 9/5 mmHg and postoperatively the PAP average dropped to 44/22 ± 14/5 mmHg. Average LOS was 21 ± 10 days.

## 4. Comment

This report describes an early experience with edge-to-edge mitral valve repair in patients undergoing LVAD implantation. The described technique offers a simple method of correcting MR. Other authors have described using an edge-to-edge repair via a transaortic approach [4, 5] and via an LV aneurysm [6].

The transapical approach described here requires only a single atrial cannulation without the need for additional dissection, cardiotomy, or application of a cross-clamp. Given its simplicity, the approach may promote a more aggressive correction of concomitant MR. This method is unlikely to be suitable for all patients. In particular, it is not suitable for patients with complex mitral valve disease. Rather it should be reserved for cases where LV dysfunction is the predominant mechanism of lacking coaptation of the mitral leaflets. Further, in our brief experience, we have repaired the valve both before and after the placement of pledgeted apical sutures, and we have found that placing the pledgets first serves to tent open the ventriculotomy and improves the exposure to the mitral valve.

Previous studies have demonstrated that MR improves with LVAD support [7]. Early findings here, however, suggest that this MVR approach may provide an even more significant reduction in MR. Thus, in early experience, edge-to-edge repair offers an efficient method of mitral valve repair in appropriate patients undergoing LVAD implantation without adding significant time or complexity to the procedure. Additional followup, however, is needed to more fully assess the long-term hemodynamic and clinical implications of the approach and to compare these findings with a control group.

## Figures and Tables

**Table 1:** Clinical and demographic characteristics of 19 patients who underwent concomitant LVAD insertion and transapical MV repair.

	*N*/average	%/std. dev.
Total	19	—
Age (years)	63.11	±8.92
Female	2	10%
White	11	58%
Black	5	26%
Asian	1	5%
Diabetes mellitus	6	32%
Hypertensive	8	42%
Previous LVAD	1	5%
Concomitant CABG	3	16%
BMI	30.02	±6.4
Current smoker	4	21%
Dialysis	1	5%
Chronic lung disease	10	53%

BMI: body mass index; CABG: coronary artery bypass graft.

**Table 2:** Preoperative and postoperative clinical characteristics of 19 patients undergoing simultaneous MV repair and LVAD insertion.

Gender	Age	CPB time (min)	Preop EF	Preop PAP average (mmHg)	Postop PAP average (mmHg)	Preop MR (1–4)	Postop MR (1–4)	Preop ET CO2 (%)	Postop ET CO2 (%)	PI	LOS
M	71	167	15%	46/31	37/25	4	0	37	26	4.7 @ 8400	33
M	62	132	21%	55/30	27/15	4	1	31	33	4 @ 9200	35
M	69	138	29%	59/26	39/18	4	0	31	31	5.3 @ 8800	24
M	61	76	17%	53/26	45/19	4	1	34	30	5.7 @ 9600	26
M	66	108	13%	47/19	53/26	4	1	30	38	3.7 @ 9600	20
M	46	92	20%	57/23	28/19	3	1	34	27	6.0 @ 9200	11
M	68	132	15%	49/20	34/16	4	1	34	32	3.0 @ 8600	35
M	42	125	21%	63/36	46/23	4	1	37	35	3.0 @ 9200	16
M	70	130	10%	55/24	33/21	4	2	36	34	5.5 @ 9600	26
F	51	121	6%	48/25	52/28	4	1	32	34	5.0 @ 8600	22
M	63	126	22%	78/39	68/31	4	1	32	32	N/A	8
M	63	248	19%	68/26	32/22	4	1	35	31	5.0 @ 8800	14
M	67	113	20%	66/31	34/19	4	2	36	40	5.0 @ 8800	Deceased in hospital: 20
M	63	102	25%	53/32	66/24	4	1	40	34	5.0 @ 9200	Deceased in hospital: 18
M	70	127	15%	N/A	N/A	4	1	N/A	N/A	6.0 @ 9200	20
M	74	141	20%	70/22	45/27	4	1	31	35	4.0 @ 9600	13
F	54	95	15%	63/31	37/20	4	1	30	28	5 @ 9200	20
M	75	148	24%	47/29	35/17	3	1	32	35	N/A	10
M	68	130	17%	55/30	75/28	4	2	36	38	6.0 @ 9200	32

CPB: cardiopulmonary bypass; EF: ejection fraction; ET CO2: end tidal CO2; LOS: length of stay; MR: mitral regurgitation; PI: pulse index; postop: postoperative; preop: preoperative.
